# Health absenteeism due to dementia in Slovenia and its costs in the period from 2015-2018

**DOI:** 10.1093/eurpub/ckac131.258

**Published:** 2022-10-25

**Authors:** M Jelenc, S Sedlak

**Affiliations:** 1 Center for Health Care, National Institute of Public Health, Ljubljana, Slovenia; 2 University of Maribor, Faculty of Health Sciences, Maribor, Slovenia

## Abstract

**Background:**

The number of employed people with certain mental or chronic illnesses is increasing. Given the fact that these are mainly diseases of the elderly and the fact that working life is increasing and populations are aging, we are already facing a public health problem that can increase without appropriate measures. Dementia mainly affects the elderly, but may occur in employed persons as well. This impairs their ability to work and is associated with costs. For the first time in Slovenia we performed a study to assess health absenteeism due to the diagnosis of dementia in the period from 2015 to 2018, to show its economic consequences and to plan the measures.

**Methods:**

The method of direct and indirect costs was used. We showed the indirect costs, which represent a loss, ie goods and services not produced on the market, namely, absenteeism due to dementia, and its economic impact. The costs of temporary absence from work were calculated on the basis of data obtained from the national health databases. The estimated cost of compensation for absence from work is based on the average gross salary. International Classification of Diseases-10 edition was used for the diagnoses of dementia; the Health Insurance Institute of Slovenia was the source of financial data.

**Results:**

Temporary absence from work due to the diagnosis of dementia in the period 2015-2018 in Slovenia amounted to around 0.6% of all calculated direct and indirect costs for this period, amounting to 11,037,275.00 EUR. The cost has been rising over the years.

**Conclusions:**

The results are underestimated, as rare cases of dementia are diagnosed before the age of 65 and due to data limitations. The employers should keep affected persons as long as possible active by adjusting the labour environment, working hours, providing an ergonomically designed workplace or by implementing preventive measures. These will reflect in positive financial effects on the economy, companies and the individuals.

**Key messages:**

• It is fundamental to encourage employers of persons with dementia to keep them in the initial phase of dementia active for as long as possible by adopting proper measures.

• Awareness of dementia is extremely important, as rapid recognition of signs of dementia and subsequent diagnosis, allow early and appropriate treatment.

